# Juices processing characteristics of Chinese bayberry from different cultivars

**DOI:** 10.1002/fsn3.778

**Published:** 2019-01-29

**Authors:** Jinhu Tian, Yanping Cao, Shiguo Chen, Zhongxiang Fang, Jianchu Chen, Donghong Liu, Xingqian Ye

**Affiliations:** ^1^ Department of Food Science and Nutrition Zhejiang Key Laboratory for Agro‐Food Processing Fuli Institute of Food Science Zhejiang R & D Center for Food Technology and Equipment Zhejiang University Hangzhou China; ^2^ Beijing Advanced Innovation Center for Food Nutrition and Human Health Beijing Technology & Business University Beijing China; ^3^ Faculty of Veterinary and Agricultural Sciences The University of Melbourne Parkville Victoria Australia

**Keywords:** bayberry juice, correlation, phenolic compounds, sensory evaluation, sugar–acid ratio

## Abstract

Fourteen cultivars of bayberry fruits were collected and used to investigate the juice processing characteristics. Results showed that bayberry juices produced from different cultivars were different in juice yield, sugar–acid ratio, phenolic compounds, and sensory quality. The highest juice yield of 84% was obtained from Zaose cultivar, and the highest total phenolic contents were observed in Lizhi juice (2243 mg/L), while Baiyangmei and Shuijing juices showed the lowest phenolic contents. Correlation analysis indicated that the sugar–acid ratio and total sugar were positively correlated with sensory preference, while titratable acidity showed a negative correlation (*p *<* *0.05). Combined with the processing characteristics and sensory preference, Wandao and Biqi were the optimal cultivars for juice processing. The research on the processing characteristics and sensory evaluation of 14 bayberry cultivars could have provided useful information on selecting suitable bayberry cultivars for juice processing.

## 1 INTRODUCTION

As one of the most economically important plants in Myricaceae family, Chinese bayberry (*Myrica rubra Sieb*. et Zucc.) has been cultivated in southern China for more than 2,000 years (Zhang et al., 2015) and the bayberry fruit is quite popular due to its attractive color (purple, red, pink, or white) and delicious taste (e.g., sweet–sour taste and pleasant aroma) (Cheng et al., 2015). It has been reported that bayberry fruits have high contents of various nutritional components, such as soluble sugars, organic acids, minerals, vitamins, and phenolic compounds and are considered to be beneficial to human health (Guo et al., 2014; Sun, Huang, Xu, Li, & Chen, 2013). However, most cultivars of bayberry fruits ripen in hot and rainy season of May and July, and the bayberry fruits decay quickly without epicarp protection (Fang et al., 2009). Xi and Zheng (1993) reported that the bayberry fruits could be stored for only 3 days at room temperature. To achieve longer time consumption, bayberry fruits are often processed into sweets, jams, wine, juice, or juice concentrate (Yu, Cai, Zhang, Feng, & Huang, 2015; Zhou et al., 2009). In addition, bayberry juice was also an important Chinese export product with a high export value Fsng & Bhandari, 2012).

Up to date, most of the researches for bayberry juice processing focused on the stability of anthocyanin, changes in antioxidants, and maintaining of flavor compounds, using different processing techniques such as ultrahigh temperature and blanching before fruit crushing as well as SO_2_ addition (Chen et al., 2013; Fang, Zhang, Sun, & Sun, 2006; Xu, Zhang, Fang, Sun, & Wang, 2014). To the best of our knowledge, few works have been reported on the processing characteristics (e.g., juice yield, sugar–acid ratio) and sensory preference of bayberry juices produced from different cultivars, which could be very important attributes for the bayberry juice industry.

In this study, fourteen cultivars of bayberry fruits that are most commonly consumed in China were selected and used to produce bayberry juice. Their processing characteristics such as juice yield, sugar contents, and sugar–acid ratio were analyzed, and their phenolic contents and sensory preferences were also studied. In addition, a correlation analysis of some physicochemical indexes of bayberry juice and its sensory preferences were also analyzed. These results may have provided some important information for food industry to select suitable bayberry fruits for juice processing.

## 2 MATERIALS AND METHODS

### 2.1 Materials

**Figure 1:**
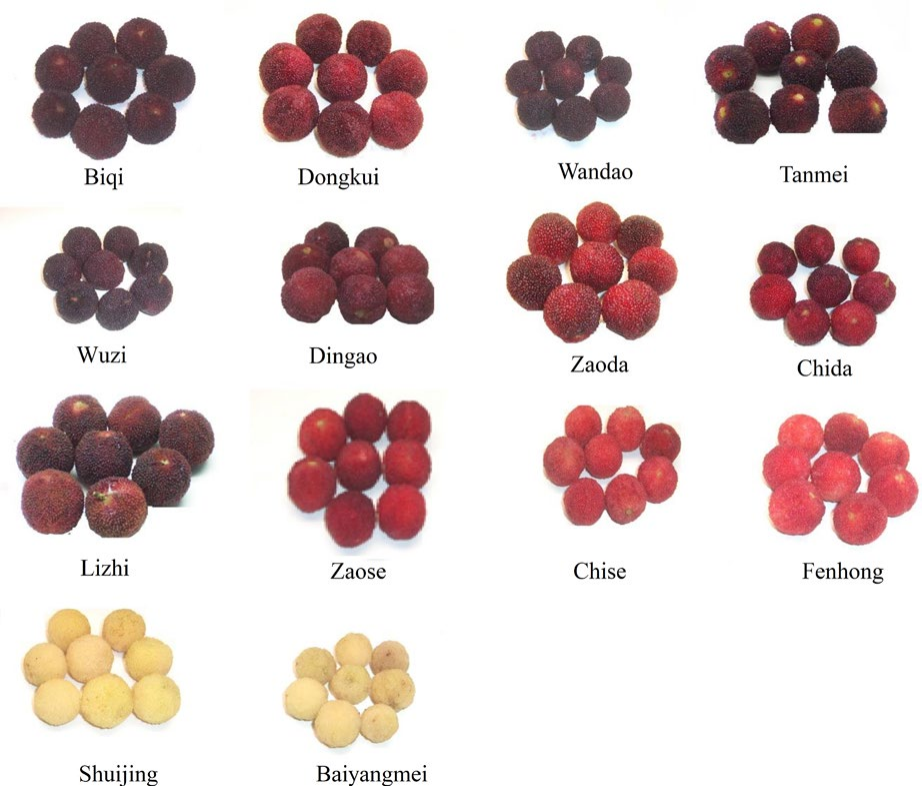
Fourteen cultivars of Chinese bayberry fruits for juice making

Fourteen cultivars of matured bayberry fruits were collected from their main production areas in Zhejiang Province, China (Figure 1). After harvesting, the bayberry fruits were stored at low temperature (4°C) and transported to our laboratory within 12 hr. Then, the bayberry juices were produced with a juicer (HR1861; Philips, China) according to its manual, and then, the juice was centrifuged, filtered, and immediately stored at −80°C for further analysis.

### 2.2 Basic parameter determination of bayberry fruits

The single fruit weight (SFW) was calculated by weighing 1,000 g bayberry fruits, and then divided by the fruit number. The juice yield was calculated by weighing the mass of 1,000 g bayberry fruits and its mass after juice processing. Ratio of fruit to kernel (RFK) was calculated by the weight of fifty fruit to its kernel. The total soluble solid (TTS) contents of the juices were measured by an Abbe refractometer (ATAGO, Japan).

### 2.3 Determination of total sugar, reducing sugar, and titratable acidity in bayberry juice

The total sugars, total reducing sugars, and the titratable acidity in bayberry juices were determined according to AOAC standard procedures (AOAC, 1995).

### 2.4 Determination of amino acids

To determine the amino acids, 5.000 g bayberry juice was accurately weighted, diluted to 100 ml with distilled water, and filtered through a 0.45‐μm filter membrane; then 5.0 ml solution was evaporated to dryness at 40°C; and 5 ml buffer solution of sodium citrate (pH 2.2) was used to dissolve the residue. The samples were analyzed with an automatic amino acid analyzer (835–50; Hitachi, Japan) according to the equipment instruction.

### 2.5 Determination of total phenolic contents

Total phenolic content was measured by the Folin–Ciocalteu method (Tian et al., 2016; Xu et al., 2008). In brief, 0.2 ml bayberry juice was mixed thoroughly with 1 ml distilled water and 1 ml Folin–Ciocalteu solution. The mixtures were kept in dark for 8 min, and then, 10 ml 17% sodium carbonate and distilled water was added to obtain a volume of 25 ml. After mixing thoroughly, the solutions were kept at room temperature for 2 hr, and the absorbance was measured at 750 nm on a spectrophotometer (UV‐2550; Shimadzu, Japan). The total phenolic content was calculated using a gallic acid standard curve (in Supporting information Figure S1) and was expressed as mg gallic acid equivalent (GAE)/L of juice.

### 2.6 Determination of total anthocyanin

The total anthocyanin content was determined by pH differential method following the procedure reported by Chen, Chen et al. (2016); Chen, Zhao et al. (2016) with some modifications. In brief, 0.025 M buffer solution of potassium chloride (pH 1.0) and 0.4 M of natrium aceticum (pH 4.5) were mixed with 1 ml bayberry juice to the volume of 10 ml, respectively. The solutions were kept in dark for 30 min, and the absorbance was measured at 510 and 700 nm on the UV‐2550 spectrophotometer, using distilled water as the blank. The anthocyanin contents were calculated as follows: C (mg/L) = V × *n* × M/ε1 × m (V represented the volume of the diluent volume, A was the absorbance of (A_520_‐A_700_) pH1–(A_520_‐A_700_) pH_4.5_, M was the molecular weight of cyanidin‐3‐glucoside, and ε1 was the molar absorptivity (29600)), Results were expressed as mg/L of cyaniding‐3‐glucoside equivalents.

### 2.7 Determination of total flavonols

Total flavonols were determined according to Capanogu, de Vos, Hall, Boyacioglu, and Beekwilder (2013) with some modification. In brief, 0.4 ml bayberry juice was added to 5 ml with ethanol, and 0.3 ml 5% sodium nitrite was added. After being kept for 6 min, 4 ml 1 M sodium hydroxide was added, and then, the volume was adjusted to 10 ml with the addition of 30% ethanol. After kept for 10 min, the absorbance was measured at 510 nm on the UV‐2550 spectrophotometer. The total flavonol concentration was calculated using a rutin standard curve (Supporting information Figure S2), and the data were expressed as mg/L rutin equivalents.

### 2.8 Sensory preference evaluation of bayberry juices

The 14 bayberry juices were placed randomly in codified cups using three‐digit code and served to each of the total of 22 panelists. The panelists were asked to grade bayberry juices for color, taste, flavor, and mouthfeel acceptability (Amerine, Pangborn, & Roessler, 1965). The detailed grade criteria were described in Supporting Information Data S1. For the preference evaluation, the bayberry juice samples were scored on a 5‐point scale from 1 (strongly dislike), 2 (dislike), 3 (neutral), 4 (like), and to 5 (strongly like) (Supporting Information Data S1).

### 2.9 Statistical analysis

All samples were prepared and analyzed in triplicate, and statistical analysis was performed using the SPSS software program (version 20.0; Chicago, USA). The Pearson correlation coefficient (R) and *p*‐value were used to show correlations and significances. Values of *p *<* *0.05 and *p *<* *0.01 were considered statistically significant and extremely significant, respectively.

## 3 RESULTS AND DISCUSSION

### 3.1 Juice processing characteristics of different bayberry cultivars

As shown in Table 1, significant differences in single fruit weight, juice yield, and ratio of fruit to kernel were observed in the 14 bayberry cultivars. The highest single fruit weight was Dongkui cultivar (22.27 g), while the lowest single fruit weight was Biqi (8.35 g). The highest juice yield was obtained from Zaose cultivar (84.00%), followed by Wandao (83.31%), Biqi (82.50%), and the lowest was from Dingao (73.50%). The highest ratio of fruit to kernel was observed in Wuzi (10.03%), and the lowest one was Biqi (6.00%).

**Table 1:** Total soluble solid, protein, sugar, and titratable acid contents of bayberry juice from different cultivars

Cultivars	SFW(g)	RFK(%)	TSS(Brix)	Juice yield (%)	Reducing sugar (g/100 ml)	Total sugar (g/100 ml)	Titratable acid (g/100 ml)	Sugar–acid ratio
Biqi	8.35 ± 0.32^f^	6.00 ± 0.17^f^	9.25 ± 0.72^de^	82.50 ± 0.30^b^	2.52 ± 0.02^fgh^	8.50 ± 0.05^cd^	0.75 ± 0.01^j^	11.38 ± 0.16^b^
Dongkui	22.27 ± 1.01^a^	7.53 ± 0.23^d^	11.25 ± 0.31^a^	74.00 ± 1.40	3.51 ± 0.11^d^	9.62 ± 0.11^a^	0.93 ± 0.01^gh^	10.41 ± 0.93^cd^
Wandao	11.68 ± 0.53^e^	7.09 ± 0.05	9.50 ± 0.20^d^	83.31 ± 0.50^a^	2.85 ± 0.10^e^	8.10 ± 0.01^e^	0.57 ± 0.01^k^	14.05 ± 0.16^a^
Tanmei	12.41 ± 0.37^d^	8.51 ± 0.33^b^	10.25 ± 0.17^bc^	76.07 ± 1.3^cd^	3.87 ± 0.03^b^	8.87 ± 0.08^b^	1.45 ± 0.02^a^	6.12 ± 0.13^gh^
Wuzi	11.90 ± 0.60	10.03 ± 0.51^a^	11.25 ± 0.33^a^	78.51 ± 0.48^cd^	3.56 ± 0.03^cd^	9.34 ± 0.10^a^	0.86 ± 0.03^hi^	10.87 ± 0.04^bc^
Dingao	12.52 ± 0.22^d^	9.50 ± 0.20^a^	8.50 ± 0.52^f^	73.50 ± 1.00^e^	2.32 ± 0.02^h^	6.55 ± 0.06^h^	1.12 ± 0.02^d^	5.85 ± 0.15^hi^
Zaoda	13.50 ± 1.13^c^	9.00 ± 0.57^b^	7.75 ± 0.30^f^	77.56 ± 0.57^d^	2.73 ± 0.05^ef^	6.23 ± 0.07^i^	0.84 ± 0.01^i^	7.74 ± 0.06^f^
Chida	10.47 ± 0.70^e^	7.52 ± 0.30^d^	10.25 ± 0.37^bc^	81.52 ± 1.20^b^	3.83 ± 0.04^bc^	8.57 ± 0.07^cd^	0.99 ± 0.01^fg^	8.68 ± 0.13^e^
Lizhi	13.56 ± 0.19^c^	9.06 ± 0.53^bc^	10.25 ± 0.10^c^	77.50 ± 2.10^cd^	4.32 ± 0.40^a^	8.42 ± 0.16^cd^	1.36 ± 0.02^b^	6.19 ± 0.13^gh^
Zaose	12.43 ± 0.32^d^	6.52 ± 0.37^e^	9.00 ± 0.10^e^	84.00 ± 0.750^a^	2.68 ± 0.06^efg^	7.59 ± 0.04^f^	1.13 ± 0.03^d^	6.70 ± 0.04^f^
Chise	11.80 ± 0.51^e^	7.00 ± 0.56^d^	11.00 ± 0.27^a^	79.53 ± 0.65^c^	2.84 ± 0.05^e^	8.73 ± 0.04^bc^	1.03 ± 0.03^ef^	8.49 ± 0.24^e^
Fenhong	14.30 ± 0.63^c^	8.03 ± 0.33^cd^	10.75 ± 0.13^ab^	79.00 ± 0.50^c^	2.87 ± 0.03^e^	9.45 ± 0.04^a^	0.96 ± 0.02^fg^	9.88 ± 0.24^d^
Shuijing	16.17 ± 0.40^b^	7.01 ± 0.71^def^	10.75 ± 0.08^ab^	75.20 ± 1.20^d^	3.47 ± 0.05^d^	8.36 ± 0.10^d^	1.08 ± 0.04^de^	7.75 ± 0.24^f^
Baiyangmei	12.05 ± 1.00^cd^	8.05 ± 0.20^c^	9.00 ± 0.20^e^	76.35 ± 1.43^d^	2.41 ± 0.12^gh^	6.85 ± 0.04^g^	1.27 ± 0.02^c^	5.38 ± 0.10^i^

*Notes*

Values in the same column sharing different superscript letters expressed as significantly different (*p *<* *0.01).

TSS, total soluble solid; SFW, single fruit weight; RFK, ratio of fruit to kernel.

### 3.2 Sugar and acid contents of bayberry juice

As shown in Table 1, the total soluble solids for these juices were ranged from 7.75 to 11.25 Brix. The highest reducing sugar was observed in Lizhi juice (4.32 g/l00 ml), and the total sugar content was in Dongkui (9.45 g/l00 ml), while the lowest was in Dingao (2.32 g/l00 ml). For the titratable acidity, the highest was observed in Lizhi (1.36 g/l00 ml) while the lowest was in Wendao juice (0.57 g/l00 ml). The sugar–acid ratio of bayberry juices from different cultivars was also calculated, and the highest sugar–acid ratio was observed in Biqi and the lowest was in Baiyangmei.

It was suggested that the content of sugar, titratable acidity, and sugar–acid ratio were the key indicators of juice quality (Amerine et al., 1965). Based on the above characteristics, hierarchical cluster analysis (HCA) was performed for these data and results showed that these bayberry cultivars could be divided into four clusters (Supporting Information Data S1). Wandao cultivar was in the first cluster with a better juice processing characteristics (higher juice yield, lower titratable acidity, higher sugar contents, and sugar–acid ratio), followed by Dongkui, Biqi, Wuzi, and Fenhong cultivars in the second cluster; Zaoda, Chida, Chise, and Shuijing cultivars were in the third cluster and Tanmei, Dingao, Lizhi, Zaose and Baiyangmei cultivars in the fourth cluster.

### 3.3 Amino acid contents of different bayberry juices

The amino acid contents of the bayberry juices produced from different cultivars are shown in Table 2. Dingao juice showed the highest total amino acids (1.672 g/L), followed by Lizhi (1.557 g/L), Baiyangmei (1.124 g/L), and Tanmei (1.167 g/L), and the lowest was observed in Dongkui juice, with only 0.057 g/L of amino acid. The profiles of amino acids from different bayberry cultivars were also different, and 13 kinds of amino acids were detected in Biqi and Lizhi cultivars, 12 kinds of amino acids were observed in Baiyangmei and Dingao, 11 kinds in Tanmei, while 10 kinds in Zaoda and Zaose. However, only six kinds of amino acids were detected in Wandao juice. In previous studies, Fang, Zhang, Tao, Sun, and Sun (2006) identified 17 kinds of amino acids in Biqi bayberry juice sediment, and Cheng, Ye, Chen, Liu, and Zhou (2008) identified 18 kinds of amino acids in the kernel of bayberry fruits. As at most only 13 amino acids were identified in this study, it is supposed that some of the amino acids might be existed as the state of protein in the bayberry juices.

**Table 2:** Amino acids of bayberry juice from different cultivars(g/L)

Cultivals	Asp	Thr	Glu	Pro	Gly	Ala	Cys	Val	Ile	Leu	Tyr	His	Arg	Total
Biqi	0.114	0.573	0.133	0.068	0.005	0.074	0.008	0.014	0.003	0.004	0.003	0.168	0.005	1.172
Dongkui	0.004	0.014	0.008	0.008	0.001	0.006	0.001	0.001	ND	ND	ND	0.014	ND	0.057
Wandao	0.009	0.026	ND	0.033	0.008	0.039	ND	ND	ND	ND	ND	0.334	0.009	0.457
Tanmei	0.069	0.432	0.109	0.264	0.006	0.083	ND	0.014	0.005	0.007	ND	0.147	0.031	1.167
Wuzi	0.014	0.032	0.014	0.131	0.007	0.021	ND	ND	ND	ND	ND	0.328	0.012	0.559
Dingao	0.13	0.867	0.105	0.188	0.005	0.086	0.002	0.017	0.005	0.008	ND	0.210	0.050	1.672
Zaoda	0.054	0.302	0.090	0.054	0.009	0.084	0.002	0.013	ND	ND	ND	0.316	0.007	0.932
Chida	0.014	0.049	0.025	0.020	0.008	0.017	ND	ND	ND	ND	0.000	0.105	0.006	0.243
Lizhi	0.114	0.638	0.137	0.344	0.005	0.094	0.004	0.014	0.005	0.005	0.012	0.159	0.026	1.557
Zaose	0.047	0.101	0.07	0.103	0.003	0.01	0.002	0.007	ND	ND	ND	0.197	0.004	0.543
Chise	0.058	0.069	0.071	0.038	0.002	0.011	ND	ND	ND	ND	ND	0.155	0.006	0.409
Fenhong	0.008	0.091	0.033	0.098	0.002	0.026	ND	0.009	ND	ND	ND	0.227	0.006	0.500
Shuijing	0.035	0.143	0.061	0.081	0.003	0.051	ND	0.007	ND	ND	ND	0.200	0.007	0.589
Baiyangmei	0.078	0.507	0.100	0.067	0.004	0.131	0.002	0.011	0.002	0.006	ND	0.206	0.010	1.124

*Note*

ND, not detected.

### 3.4 Phenolic compounds in bayberry juices

**Figure 2:**
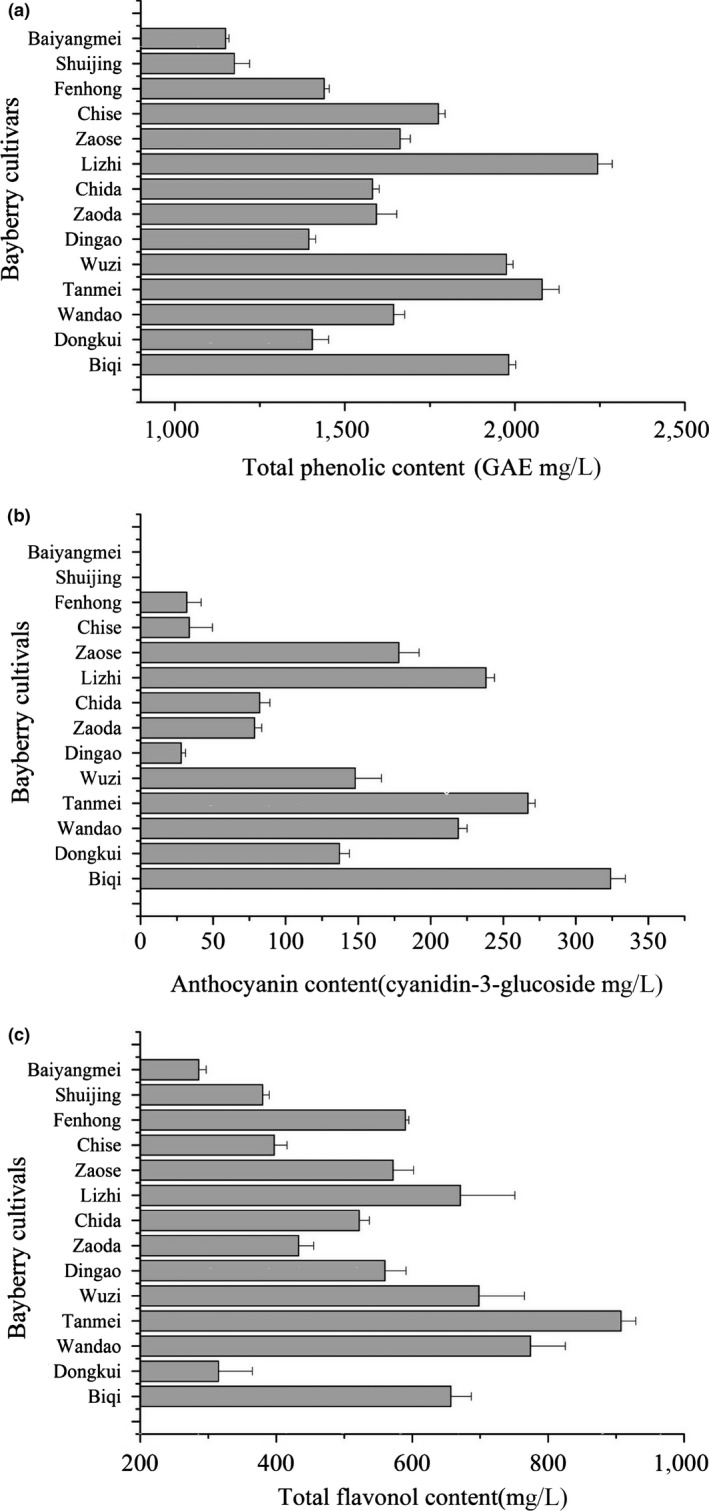
Total phenolic, anthocyanin, and flavonol contents in bayberry juices(1, total phenolic; 2, anthocyanin; 3, flavonol)

Attributing to their bioactive functionalities, phenolic compounds derived from fruits have received more and more attention (Shahidi & Ambigaipalan, 2015). There is evidence that the strong antioxidant capacity of bayberry fruits (Chen, Chen et al. (2016); Chen, Zhao et al. (2016)), freeze‐dried powder (Fang & Bhandari, 2011), juice (Fang et al., 2009), and pomace (Zhou et al., 2009) is highly correlated with their phenolic content. The total contents of phenolics, anthocyanins, and flavonoids in bayberry juice from different cultivars are shown in Figure 2. The highest total phenolic contents were observed in Lizhi juice (2,243 mg/L), followed by Tanmei (2,080 mg/L), Wuzi (1,975 mg/L), and Biqi (1,982 mg/L), and the lowest two were in Shuijing and Baiyangmei, which were about 1,175 and 1,149 mg/L, respectively. Similar results were also observed by Cheng et al. (2016). In terms of total anthocyanins, Biqi juice had the highest anthocyanin contents (324 mg/L), followed by Tanmei (267 mg/L) and Lizhi (238 mg/L). Little anthocyanin was detected in the juices of Baiyangmei and Shuijing, which were classified as white‐colored bayberry cultivars and planted only in a few orchards because of their low production yields (Fang et al., 2009). For total flavonoid contents, the highest was observed in Tanmei juice (907 mg/L), followed by Wendao (774 mg/L), Lizhi (671 mg/L), and Biqi (657 mg/L), and the lowest one was observed in Baiyangmei with 286 mg/L.

### 3.5 Sensory evaluation of bayberry juices

The sensory evaluations of bayberry juices produced from different cultivars are shown in Table 3. Based on color, taste, flavor, and shape, Wandao juice showed the highest sensory score (91.0), followed by Biqi (89.2) and Tanmei (86.0), while Dingao, Zaodao, and Zaose showed quite lower scores of 76.9, 77.8, and 78.7, respectively. The preference evaluation showed that Wandao received the highest score (4.2), followed by Chida (3.9) and Biqi (3.6). The two lowest preference scores were observed in Baiyangmei (2.2) and Dingao (2.1), which indicated that the juices made from these cultivars were not acceptable. In general, combined with the sensory and preference evaluation, the bayberry juice made from Wandao cultivar was the best in this study, followed by Biqi and Chida. In a previous study, Cheng et al. (2016) also made a comparative sensory analysis of 11 bayberry juices and reported that a relatively higher sensory score of Wandao and Biqi cultivar than other cultivars.

**Table 3:** Analysis of sensory and preference evaluation of bayberry juices from different cultivars

Cultivars	Color	Taste	Flavor	Shape	Total	Preference
Biqi	28.0 ± 1.5^b^	26.6 ± 1.5^ab^	16.9 ± 2.2^ab^	17.7 ± 2.2^ab^	89.2 ± 1.5^ab^	3.6 ± 0.7^abc^
Dongkui	24.0 ± 1.4^f^	25.3 ± 1.5^bcd^	16.8 ± 2.4^ab^	16.3 ± 2.0^abcd^	82.4 ± 1.5^cde^	2.9 ± 1.2^cdefg^
Wandao	27.0 ± 1.8^cd^	27.9 ± 1.5^a^	18.0 ± 1.9^a^	18.1 ± 1.6^a^	91.0 ± 1.^5a^	4.2 ± 0.9^a^
Tanmei	28.6 ± 1.5^b^	24.1 ± 1.5^cde^	16.8 ± 2.0^abc^	16.5 ± 1.7^abcd^	86.0 ± 1.5^abc^	2.7 ± 0.9^efg^
Wuzi	26.4 ± 2.1^de^	25.1 ± 1.5^cd^	16.5 ± 2.6^abcd^	16.4 ± 2.1^bc^	84.5 ± 1.5^bcd^	3.3 ± 1.1^bcde^
Dingao	24.4 ± 1.5^f^	22.7 ± 1.5^e^	16.0 ± 2.4^abc^	13.8 ± 2.7^f^	76.9 ± 1.5^f^	2.1 ± 0.9^g^
Zaodao	23.6 ± 1.3^f^	23.8 ± 1.5^de^	15.5 ± 2.3^bc^	14.9 ± 2.4^def^	77.8 ± 1.5^ef^	2.5 ± 0.9^efg^
Chida	25.6 ± 1.6^e^	26.5 ± 1.5^abc^	16.6 ± 1.6^ab^	16.8 ± 2.2^bc^	85.5 ± 1.5^bc^	3.9 ± 0.8^ab^
Lizhi	27.7 ± 1.5^bc^	24.7 ± 1.5^cde^	17.0 ± 2.1^ab^	16.5 ± 1.7^abcd^	85.9 ± 1.5^abc^	2.6 ± 0.9^defg^
Zaose	24.1 ± 1.9^f^	24.3 ± 1.5^cde^	14.9 ± 2.1^bc^	15.5 ± 1.7c^def^	78.7 ± 1.5^ef^	2.6 ± 1.0^defg^
Chise	21.4 ± 1.1^g^	25.2 ± 1.5^bcd^	16.2 ± 3.5^abc^	16.4 ± 1.9^abcd^	79.2 ± 1.5^def^	3.3 ± 0.8^bcde^
Fenhong	22.2 ± 1.7^g^	25.7 ± 1.5^bcd^	15.7 ± 2.6^bc^	16.5 ± 2.4a^bcd^	80.1 ± 1.5^def^	3.4 ± 1.0^bcd^
Shuijing	27.0 ± 0.0^cd^	23.8 ± 1.5^de^	14.2 ± 3.1^c^	15.8 ± 2.2^bcde^	80.8 ± 1.5^cdef^	3.1 ± 1.4^cdef^
Baiyangmei	30.0 ± 0.0^a^	22.8 ± 1.5^e^	14.8 ± 2.6^bc^	14.4 ± 2.4^ef^	82.0 ± 1.5^cdef^	2.2 ± 0.8^fg^

*Note*

Values in the same column sharing different Superscript letters expressed as significantly different (*p *<* *0.01).

### 3.6 Correlation analysis of some physicochemical indexes of bayberry juice and its sensory preference

Correlation coefficients of total soluble solid, reducing sugar, total sugar, titratable acidity, sugar–acid ratio, and sensory preference are shown in Table 4. The sensory preference correlated extremely highly (*p *<* *0.01) with sugar–acid ratio (*p *<* *0.01), and significantly with total sugar (*p *<* *0.05), while the titratable acidity showed a negative correlation with the sensory preference (*p *<* *0.05). The juices produced from Dingao, Lizhi, and Baiyangmei had the lowest sensory preference score, which might partially due to their lower contents of total sugar and sugar–acid ratio. The results suggested that bayberry juices should be produced from higher sugar–acid ratio cultivars to meet consumer demands.

**Table 4:** Correlation coefficients of total soluble solid, total sugar, reducing sugar, titratable acid, sugar–acid ratio to preference of bayberry juice

	Total soluble solid	Reducing sugar	Total sugar	Sugar–acid ratio	Preference
Titratable acid	0.062	0.356	‐0.082	‐0.844**	‐0.556*
Total soluble solid		0.603*	0.917**	0.473	0.537*
Reducing sugar			0.562*	‐0.052	0.236
Total sugar				0.473	0.537*
Sugar–acid ratio					0.705**

*Indicates significant difference at the level of 0.05, while; **indicates significant difference at the level of 0.01.

## 4 CONCLUSION

The present study demonstrated that the juice processing characteristics of 14 bayberry cultivars were different in juice yield, total sugar content, and sugar–acid ratio. In addition, the phenolic contents from white cultivar juices (Baiyangmei and Shuijing) showed little anthocyanin and a much lower total phenolic contents than the purple and red cultivars. From the perspective of health benefits, the purple and red bayberry cultivars may be a better choice for juice processing (Sun et al., 2013). In combination of juice processing characteristics and sensory preference evaluation, this study suggested that Wandao cultivar was the best for bayberry juice processing, followed by Biqi cultivar.

## CONFLICT OF INTEREST

The authors declare that they have no conflict of interest.
