# Supplementary material for: Juices processing characteristics of Chinese bayberry from different cultivars
Source: Food Sci Nutr. 2019 Jan 29;7(2):404–11. doi: 10.1002/fsn3.778 (PMC6392854; doi:10.1002/fsn3.778)
Supplement: Supplementary file 1 [file FSN3-7-404-s001.docx]

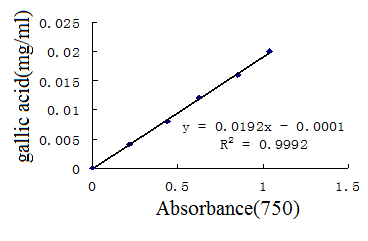


Figure 1 Standard curve for determination of total phenolic


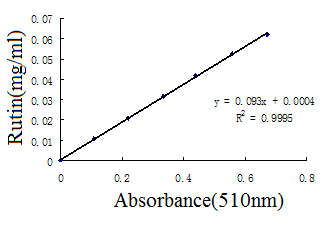


Figure 2 Standard curve for determination of total flavonoid


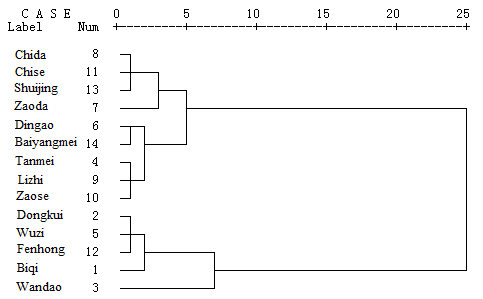


Figure 3 Hierarchical cluster analyses for bayberry juice from different cultivars

Table 1 Scoring criteria for bayberry juice

| Grade | Color | Taste | Flavor | Mouthfeel | Total |
| --- | --- | --- | --- | --- | --- |
|  | 30 | 30 | 20 | 20 | 100 |
| First level | 27-30 | 27-30 | 18-20 | 18-20 | 90-100 |
|  | Bright red, purple | Typical taste of bayberry juice, sweet and sour taste, no objectional odor | Typical flavor of bayberry juice | Good mouthfeel  harmonious styles |  |
| Second level | 24-27 | 24-27 | 15-18 | 15-18 | 78-90 |
|  | Relatively bright red, Relatively purple | Common taste of bayberry juice, relatively sweet and sour taste,  no objectional odor | Relatively typical flavor of bayberry juice | Relatively Good mouthfeel  and harmonious styles |  |
| Third level | 20-24 | 20-24 | 10-15 | 10-15 | 46-78 |
|  | Red or yellow | Atypical taste of bayberry juice, unfavorable sweet and sour, objectional odor | Inconspicuous of bayberry juice | Bad mouthfeel |  |

Supplement: Scoring criteria for the color of Baiyangmei and Shuijing

| Grade | First level | Second level | Third level |
| --- | --- | --- | --- |
|  | Closely to the natural color of bayberry fruit | Faint yellow | More yellow in color |
| scores | 27-30 | 24-26 | 20-23 |

Table 2 Scoring criteria of preference of bayberry juice

| strongly dislike | dislike | neutral | like | strongly like |
| --- | --- | --- | --- | --- |
| 1 | 2 | 3 | 4 | 5 |
